# Supplementary material for: A Hydrofluoric Acid-Free Green Synthesis of Magnetic M.Ti2CTx Nanostructures for the Sequestration of Cesium and Strontium Radionuclide
Source: Nanomaterials (Basel). 2022 Sep 19;12(18):3253. doi: 10.3390/nano12183253 (PMC9502560; doi:10.3390/nano12183253)
Supplement: Supplementary file 1 [file nanomaterials-12-03253-s001.zip › nanomaterials-1871641-supplementary.pdf]

Supplementary Information

A Hydrofluoric Acid-Free Green Synthesis of Magnetic M.Ti_2CT_x Nanostructures for the Sequestration of Cesium and Strontium Radionuclide

Jibran Iqbal [1,*], Kashif Rasool [2], Fares Howari [1], Yousef Nazzal [1], Tapati Sarkar [3] and Asif Shahzad [3,*]

[1] College of Natural and Health Sciences, Zayed University, Abu Dhabi 144534, United Arab Emirates,
Fares.Howari@zu.ac.ae (F.H.); Yousef.Nazzal@zu.ac.ae (Y.N.)

[2] Qatar Environment and Energy Research Institute, Hamad Bin Khalifa University (HBKU), Qatar
Foundation, P.O. Box 5824, Doha, Qatar; kashifrsl@hbku.edu.qa

[3] Department of Materials Science and Engineering, Uppsala University, Box 35,
SE-75103 Uppsala, Sweden; tapati.sarkar@angstrom.uu.se

* Correspondence: Jibran.iqbal@zu.ac.ae (J.I.); asif.shahzad@angstrom.uu.se (A.S.);
Tel.: +971-55-9188346 (J.I.); +46-764510726 (A.S.)

Supplementary Note

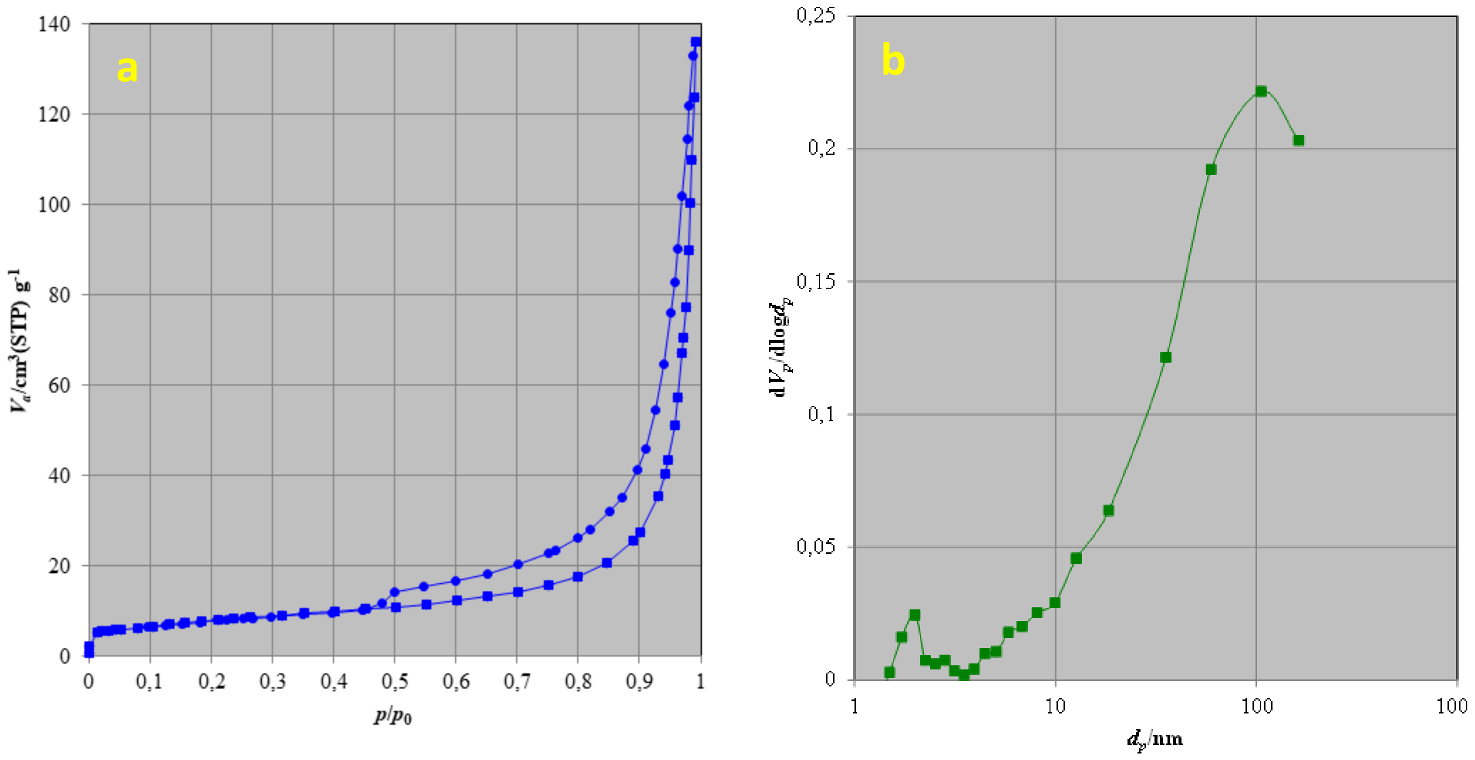

Figure S1. (a) The BET adsorption/desorption isotherm and (b) BHJ pore size distribution graph of *M.Ti$_2$CT$_x$.A$_{III}$*

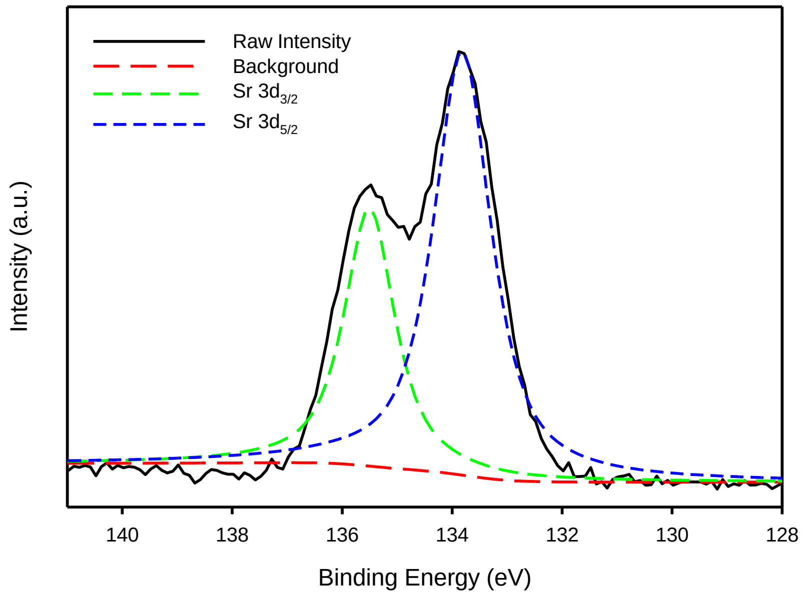

Figure S2. The XPS peak fitting analysis of Sr 3d in *M.*Ti_2CT_x*.*A_{III} after radionuclides adsorption

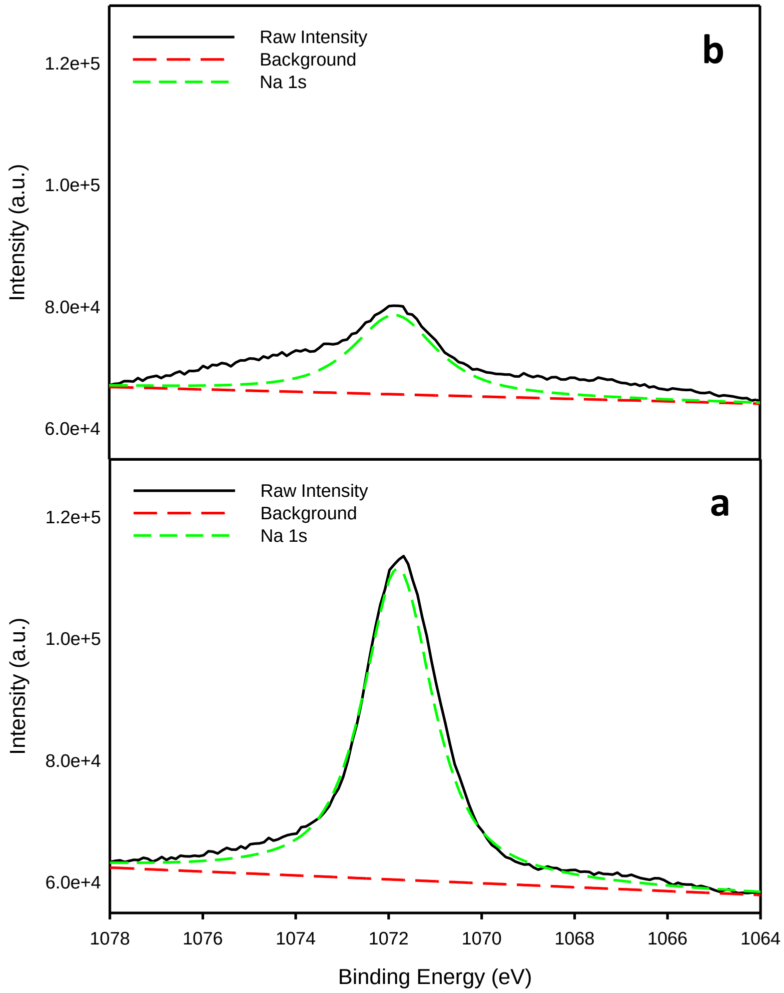

Figure S3. The XPS peak fitting analysis of Na 1s (a) before and (b) after radionuclides adsorption in *M.*Ti_2CT_x*.*A_{III}

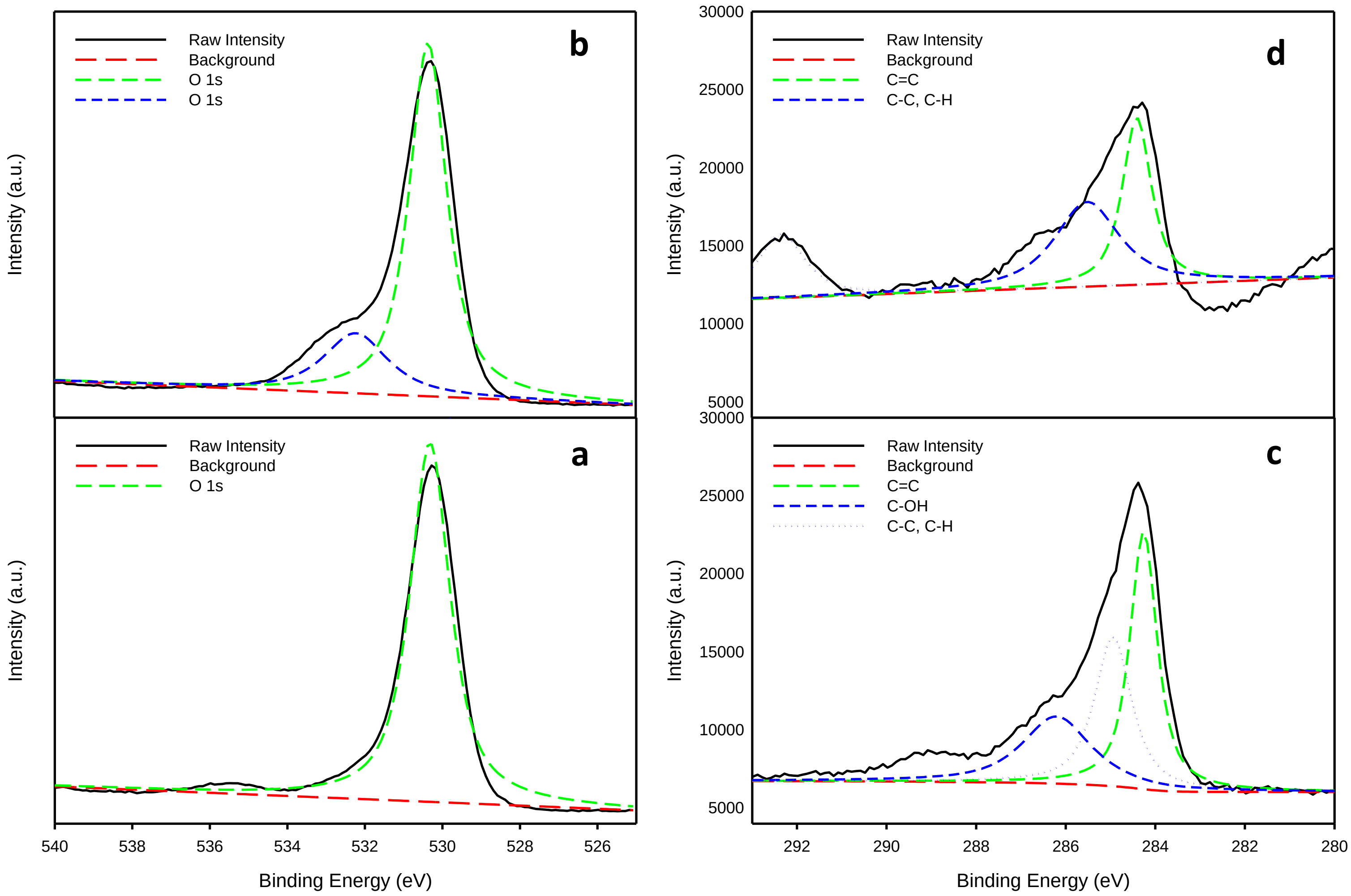

Figure S4. The XPS peak fitting analysis of O 1s and C 1s before (a & c) and after (b & d) radionuclides adsorption, respectively, in *M.*Ti_2CT_x*.*A_{III}

Table S1. Elemental composition of $M.Ti_2CT_x.A_{III}$ measured in SEM-EDS analysis.

Element	Line Type	Wt%	Wt% Sigma	Atomic %
C	K series	31.62	0.21	47.21
O	K series	33.83	0.25	37.91
Na	K series	5.13	0.07	4.00
Al	K series	0.12	0.03	0.08
Ti	K series	26.17	0.17	9.80
Fe	K series	3.13	0.11	1.01
Total:		100.00		100.00

Table S2. Elemental composition of $M.Ti_2CT_x.A_{III}$ after Sr^{2+} and Cs^{+} adsorption, measured in SEM-EDS analysis.

Element	Line Type	Wt%	Wt% Sigma	Atomic %
C	K series	21.91	0.18	37.04
O	K series	35.28	0.21	44.78
Na	K series	1.20	0.04	1.06
Al	K series	0.58	0.03	0.44
Si	K series	1.88	0.05	1.36
Ti	K series	28.80	0.16	12.21
Fe	K series	6.27	0.12	2.28
Sr	L series	2.57	0.12	0.59
Cs	L series	1.52	0.14	0.23
Total:		100.00		100.00

Table S3. Elemental composition (Atomic%) of as-prepared *M.*$Ti_2CT_x.A_{III}$ and after Sr^{2+} and Cs^+ adsorption, measured in XPS analysis.

Sample	Atomic concentration (%)							
	**Ti 2p3**	**C 1s**	**Na 1s**	**O 1s**	**Al 2p**	**Fe 2p**	**Sr 3d**	**Cs 3d**
M.$Ti_2CT_x.A_{III}$	21.89	16	8.54	52.44	1.12	–	–	–
Sr^{2+},Cs^+@*M.*$Ti_2CT_x.A_{III}$	22.02	15.8	1.51	59.54	–	–	1.74	–
